# Screening for Social Determinants of Health During Primary Care and Emergency Department Encounters

**DOI:** 10.1001/jamanetworkopen.2023.48646

**Published:** 2023-12-19

**Authors:** Stacie Vilendrer, Samuel C. Thomas, Tom Belnap, Kim Burnisholz, Nancy Song, Raj Srivastava, Sara Singer

**Affiliations:** 1 Division of Primary Care and Population Health, Department of Medicine, Stanford University School of Medicine, Stanford, California; 2 Healthcare Delivery Institute, Intermountain Health, Murray, Utah

## Abstract

This cross-sectional study explores characteristics of patients who are screened and who screen positive for social determinants of health (SDOH) needs in different clinical settings within a large integrated health system.

## Introduction

The Centers for Medicare & Medicaid Services have mandated inpatient screening of selected social determinants of health (SDOH) needs starting in 2024.^1^ However, wide scale screening of SDOH has not become standard practice, in part due to limited resources.^2^ The optimal clinical setting(s) for deployment of SDOH resources to improve health outcomes is uncertain.^3^ This study aims to explore characteristics of patients who are screened and who screen positive for SDOH needs in different clinical settings within a large integrated health system.

## Methods

This cross-sectional study was approved by the Intermountain Health institutional review board (IRB) and Stanford Medicine IRB. The Intermountain Health IRB determined this study to be exempt from informed consent as it examined previously collected data used during patient care. We followed the STROBE reporting guideline.

Between September 1, 2019, and December 31, 2020, Intermountain Healthcare primary care clinics and emergency department (ED) deployed systemwide standardized SDOH screens (eAppendix in Supplement 1).^4^ Electronic health record data from Intermountain Health were used to determine overall and positive SDOH screening rates and patient characteristics from encounters in 3 settings: in-person primary care, virtual primary care, and the ED. To compare screening across settings, we performed multivariable regressions using SAS version 9.4 (SAS Institute), with being screened and screening positive for 1 or more SDOH needs as 2 dependent variables; and patient demographics, social status, clinical status, health care utilization, and area deprivation index, a proxy for residents’ socioeconomic status,^5^ as independent variables. One-sided *P* < .05 was considered statistically significant. Statistical analysis was performed from September 2019 to November 2023.

## Results

Among all patients in the 2 832 221 encounters conducted by Intermountain Health during the study period, 19 343 (0.7%) were American Indian or Alaska Native, 39 599 (1.4%) were Asian, 29 842 (1.1%) were Black or African American, 260 076 (9.2%) were Hispanic, 31 729 (1.1%) were Native Hawaiian or Pacific Islander, 2 637 796 (93.2%) were White, and 72 802 (2.60%) were other or unknown race and ethnicity; 1 602 471 (56.6%) were female; and mean (SD) age was 45.5 (24.5) years. Among 2 058 836 in-person primary care encounters, 343 392 (16.7%) were screened for SDOH needs; among 389 789 virtual primary care encounters, 31 297 (8.0%) were screened; and among 383 596 ED encounters, 934 (0.2%) were screened (Table 1). Patients seen during ED encounters were more likely to be unmarried (60.2% [230 887 of 383 596]) than the overall study population (51.1% [1 446 188 of 2 832 221]). ED encounters were significantly more likely to reveal SDOH needs relative to all encounters (51.6% [482 of 934] vs 7.8% [29 394 of 375 623]; *P* < .001) despite significantly lower screening rates (0.2% [934 of 383 596] vs 13.3% [375 623 of 2 832 221]; *P* < .001). Among included patient characteristics, married marital status and diagnoses of chronic pulmonary disease or cancer were significantly less likely to be associated with positive SDOH screening (Table 2). Hispanic ethnicity, highest area deprivation index, and diagnosis of depression, drug use disorder, or obesity were significantly associated with having a positive SDOH screen (Table 2).

**Table 1.** Social Determinants of Health Screening Completion and Results Across Various Sites of Service

Variables	Encounters, No. (%)			
	All (n = 2 832 221)	In-person primary care (n = 2 058 836)	Scheduled video visit (n = 389 789)	Emergency department (n = 383 596)
SDOH screening status				
Not screened	2 456 598 (86.7)	1 715 444 (83.3)	358 492 (92.0)	382 662 (99.8)
Screened	375 623 (13.3)	343 392 (16.7)	31 297 (8.0)	934 (0.2)
Type of SDOH screening tool				
SEEK	21 291 (5.7)	21 228 (6.2)	51 (0.2)	12 (1.3)
Social check (ie, PRAPARE lite)	354 332 (94.3)	322 164 (93.8)	31 246 (99.9)	922 (98.7)
Positive for SDOH needs^a^				
Yes	29 394 (7.8)	26 710 (7.8)	2202 (7.0)	482 (51.6)
No	346 229 (92.2)	316 682 (92.2)	29 095 (93.0)	452 (48.4)

Abbreviations: PRAPARE, Protocol for Responding to and Assessing Patients’ Assets, Risks, and Experiences; SDOH, social determinants of health; SEEK, Safe Environment for Every Kid.

^a^ Percentage positive for SDOH needs is based on the total number screened.

**Table 2.** Patient Characteristics and Logistic Regression Modeling for Screening Completion and Those That Screen Positive for SDOH Needs Stratified by Site of Service

Characteristics	All patient encounters			In-person primary care encounters			Scheduled video visit encounters			Emergency department encounters		
	No. (%) (N = 2 832 221)	OR (95% CI)		No. (%) (n = 2 058 836)	OR (95% CI)		No. (%) (n = 389 789)	OR (95% CI)		No. (%) (n = 383 596)	OR (95% CI)	
		Screened for SDOH (n = 375 623)	Positive for SDOH needs (n = 29 394)		Screened for SDOH (n = 343 392)	Positive for SDOH needs (n = 26 710)		Screened for SDOH (n = 31 297)	Positive for SDOH needs (n = 2202)		Screened for SDOH (n = 934)	Positive for SDOH needs (n = 482)
Patient demographics												
Age group, y												
0-4 (vs ≥18)	84 147 (3.0)	1.22 (1.19-1.24)	4.95 (4.72-5.19)	69 570 (3.4)	1.17 (1.14-1.19)	4.66 (4.44-4.89)	2139 (0.6)	0.15 (0.11-0.22)	0.72 (0.34-1.52)	12 438 (3.2)	0.92 (0.53-1.58)	0.55 (0.20-1.51)
5-17 (vs ≥18)	425 735 (15.0)	0.92 (0.91-0.93)	1.02 (0.98-1.06)	320 981 (15.6)	0.93 (0.91-0.94)	1.04 (1.0-1.08)	52 087 (13.4)	0.18 (0.17-0.19)	0.16 (0.12-0.22)	52 667 (13.7)	0.89 (0.69-1.17)	0.80 (0.54-1.19)
Male (vs female)	1 229 750 (43.4)	1.04 (1.03-1.05)	1.03 (1.00-1.05)	905 564 (43.0)	1.09 (1.08-1.09)	1.07 (1.04-1.09)	152 199 (39.1)	0.91 (0.89-0.93)	0.85 (0.77-0.93)	171 987 (44.8)	0.80 (0.70-0.92)	0.89 (0.73-1.09)
Married (vs not married)	1 386 033 (48.9)	1.15 (1.14-1.16)	0.57 (0.56-0.59)	1 039 760 (50.5)	1.07 (1.06-1.08)	0.54 (0.52-0.55)	193 564 (49.7)	1.11 (1.08-1.13)	0.61 (0.56-0.67)	152 709 (39.8)	0.52 (0.44-0.61)	0.47 (0.37-0.60)
Race^a^												
Minoritized race (combined in regression)	193 315 (6.8)	NA	NA	133 941 (6.5)	NA	NA	22 086 (5.7)	NA	NA	37 288 (9.7)	NA	NA
American Indian or Alaska Native	19 343 (0.7)	NA	NA	12 513 (0.6)	NA	NA	2032 (0.5)	NA	NA	4798 (1.3)	NA	NA
Asian	39 599 (1.4)	NA	NA	31 549 (1.5)	NA	NA	4457 (1.1)	NA	NA	3593 (0.9)	NA	NA
Black or African American	29 842 (1.1)	NA	NA	19 156 (0.9)	NA	NA	3860 (1.0)	NA	NA	6826 (1.8)	NA	NA
Native Hawaiian/Pacific Islander	31 729 (1.1)	NA	NA	21 628 (1.1)	NA	NA	3294 (0.9)	NA	NA	6807 (1.8)	NA	NA
Other/unknown^b^	72 802 (2.6)	NA	NA	49 095 (2.4)	NA	NA	8443 (2.2)	NA	NA	15 264 (4.0)	NA	NA
White	2 637 796 (93.2)	1.07 (1.05-1.09)	0.80 (0.77-0.84)	1 923 880 (93.5)	1.02 (1.01-1.04)	0.77 (0.74-0.81)	367 665 (94.3)	0.98 (0.93-1.03)	0.71 (0.62-0.83)	346 251 (90.3)	1.28 (1.00-1.63)	1.10 (0.80-1.52)
Hispanic (vs non-Hispanic)	260 076 (9.2)	0.88 (0.87-0.90)	1.19 (1.14-1.23)	175 489 (8.5)	0.94 (0.92-0.95)	1.24 (1.19-1.29)	29 123 (7.5)	0.95 (0.91-1.01)	1.19 (1.02-1.39)	55 464 (14.5)	1.86 (1.58-2.20)	1.91 (1.52-2.40)
Preferred language English (vs other)	2 763,671 (97.6)	1.53 (1.48-1.57)	1.10 (1.02-1.19)	2 012 947 (97.8)	1.37 (1.33-1.41)	0.98 (0.91-1.07)	384 487 (98.6)	1.31 (1.17-1.46)	0.83 (0.60-1.15)	366 237 (95.5)	2.29 (1.44-3.64)	2.71 (1.32-5.59)
Patient social considerations												
Area deprivation index												
Least deprived	616 633 (21.8)	NA	NA	454 708 (22.1)	NA	NA	96 544 (24.8)	NA	NA	65 381 (17.0)	NA	NA
Fourth-most deprived	694 863 (24.5)	0.95 (0.95-0.96)	1.13 (1.08-1.17)	512 781 (24.9)	0.97 (0.96-0.99)	1.14 (1.09-1.18)	101 006 (25.9)	0.91 (0.88-0.94)	1.07 (0.94-1.23)	81 076 (21.1)	1.73 (1.28-2.35)	1.66 (1.08-2.56)
Third-most deprived	574 581 (20.3)	0.85 (0.84-0.86)	1.33 (1.28-1.39)	420 219 (20.4)	0.89 (0.88-0.90)	1.37 (1.31-1.43)	78 097 (20.0)	0.78 (0.75-0.81)	1.19 (1.04-1.37)	76 265 (19.9)	1.59 (1.17-2.17)	1.42 (0.91-2.23)
Second-most deprived	488 558 (17.3)	0.81 (0.80-0.82)	1.48 (1.42-1.54)	353 945 (17.2)	0.86 (0.85-0.87)	1.53 (1.46-1.59)	60 204 (15.5)	0.82 (0.79-0.85)	1.52 (1.32-1.75)	74 409 (19.4)	1.48 (1.09-2.02)	1.49 (0.96-2.31)
Most deprived	457 586 (16.2)	0.79 (0.78-0.80)	1.81 (1.74-1.88)	317 183 (15.4)	0.86 (0.85-0.87)	1.88 (1.80-1.96)	53 938 (13.8)	0.84 (0.81-0.87)	1.84 (1.60-2.10)	86 465 (22.5)	3.98 (3.03-5.25)	4.16 (2.82-6.14)
Rural (vs urban)	213 453 (7.5)	0.93 (0.92-0.94)	0.82 (0.79-0.87)	163 662 (8.0)	0.91 (0.90-0.92)	0.79 (0.75-0.83)	22 222 (5.7)	1.14 (1.09-1.19)	1.09 (0.91-1.31)	27 569 (7.2)	0.55 (0.39-0.78)	0.78 (0.51-1.20)
Clinical status												
Elixhauser chronic conditions												
Psychoses	118 756 (4.2)	0.78 (0.76-0.80)	1.02 (0.97-1.07)	66 940 (3.3)	0.91 (0.89-0.93)	1.19 (1.13-1.25)	25 145 (6.5)	0.58 (0.55-0.62)	0.81 (0.69-0.95)	26 671 (7.0)	0.91 (0.75-1.10)	0.99 (0.78-1.28)
Depression	1 205 067 (42.6)	1.07 (1.06-1.08)	2.09 (2.03-2.15)	804 305 (39.1)	1.09 (1.08-1.10)	2.21 (2.15-2.28)	242 465 (62.2)	0.74 (0.72-0.76)	1.79 (1.60-2.00)	158 297 (41.3)	1.81 (1.53-2.15)	1.78 (1.39-2.27)
Drug use disorder	281 100 (9.9)	0.71 (0.70- 0.72)	1.33 (1.28-1.38)	155 987 (7.6)	0.84 (0.83- 0.86)	1.56 (1.50-1.62)	53 953 (13.8)	0.69 (0.66- 0.72)	1.27 (1.14-1.41)	71 160 (18.6)	1.94 (1.66-2.26)	2.57 (2.07-3.18)
Alcohol use disorder	157 607 (5.6)	0.84 (0.83-0.86)	1.13 (1.08-1.18)	89 904 (4.4)	0.95 (0.93-0.97)	1.26 (1.20-1.32)	26 962 (6.9)	0.88 (0.84-0.93)	1.07 (0.92-1.23)	40,741 (10.6)	1.20 (1.01-1.42)	1.05 (0.83-1.32)
Kidney failure	383 069 (13.5)	1.14 (1.13-1.16)	0.90 (0.86-0.94)	298 374 (14.5)	1.07 (1.06-1.09)	0.85 (0.81-0.90)	46 510 (11.9)	1.19 (1.14-1.24)	0.98 (0.84-1.14)	38 185 (10.0)	0.58 (0.44-0.76)	0.46 (0.31-0.69)
Peripheral vascular disorders	383 370 (13.5)	0.94 (0.93-0.96)	0.94 (0.90-0.98)	296 819 (14.4)	0.89 (0.88-0.90)	0.89 (0.85-0.93)	46 734 (12.0)	1.01 (0.97-1.05)	0.99 (0.87-1.14)	39 817 (10.4)	1.01 (0.81-1.25)	0.95 (0.69-1.29)
Liver disease	410 578 (14.5)	0.96 (0.95-0.97)	1.11 (1.07-1.14)	290 676 (14.1)	0.97 (0.96-0.98)	1.10 (1.06-1.14)	60 849 (15.6)	0.94 (0.91-0.97)	1.02 (0.91-1.14)	59 053 (15.4)	1.69 (1.45-1.97)	1.99 (1.61-2.45)
Hypertension, uncomplicated	1 207 826 (42.7)	1.13 (1.12-1.14)	0.97 (0.94-0.99)	909 231 (44.2)	1.05 (1.04-1.06)	0.88 (0.85-0.91)	158 877 (40.8)	1.29 (1.25-1.32)	1.19 (1.07-1.32)	139 718 (36.4)	0.89 (0.76-1.05)	0.77 (0.61-0.96)
Hypertension, complicated	353 212 (12.5)	0.80 (0.79-0.81)	0.90 (0.85-0.95)	271 115 (13.2)	0.81 (0.79-0.82)	0.91 (0.86-0.97)	42 050 (10.8)	0.88 (0.84-0.93)	0.90 (0.75-1.08)	40 047 (10.4)	1.36 (1.02-1.83)	1.21 (0.79-1.85)
Diabetes^c^	585 327 (20.7)	0.99 (0.98-1.00)	1.18 (1.14-1.22)	440 828 (21.4)	0.96 (0.95-0.97)	1.15 (1.11-1.19)	80 094 (20.6)	0.95 (0.92-0.98)	1.12 (1.01-1.25)	64 405 (16.8)	0.96 (0.81-1.15)	1.11 (0.88-1.41)
Obesity	887,212 (31.3)	1.07 (1.07-1.08)	1.31 (1.27-1.35)	642 069 (31.2)	1.04 (1.03-1.04)	1.27 (1.23-1.31)	141,336 (36.3)	0.93 (0.91-0.96)	1.17 (1.06-1.29)	103 807 (27.1)	1.54 (1.33-1.80)	1.69 (1.37-2.09)
Chronic pulmonary disease	1 161 305 (41.0)	0.98 (0.97-0.99)	0.88 (0.86-0.90)	838 288 (40.7)	1.02 (1.01-1.03)	0.90 (0.88-0.93)	169 640 (43.5)	0.89 (0.87-0.91)	0.80 (0.73-0.87)	153 377 (40.0)	0.71 (0.61-0.82)	0.74 (0.60-0.91)
Cardiovascular disease^d^	303 925 (10.7)	0.72 (0.71-0.73)	0.78 (0.74-0.82)	232 473 (11.3)	0.69 (0.68-0.70)	0.73 (0.69-0.77)	36 793 (9.4)	0.85 (0.81-0.89)	1.04 (0.88-1.22)	34 659 (9.0)	0.83 (0.64-1.08)	0.85 (0.59-1.24)
Cancer^e^	290 106 (10.2)	0.94 (0.93-0.96)	0.75 (0.72-0.79)	222 433 (10.8)	0.92 (0.91-0.94)	0.74 (0.70-0.77)	37 687 (9.7)	0.91 (0.88-0.95)	0.73 (0.62-0.85)	29 986 (7.8)	0.73 (0.55-0.95)	0.60 (0.40-0.90)
Other neurological disorders	415 452 (14.7)	0.79 (0.78-0.80)	0.94 (0.91-0.98)	278 696 (13.5)	0.83 (0.82-0.84)	0.99 (0.96-1.03)	67 510 (17.3)	0.73 (0.71-0.76)	0.86 (0.77-0.96)	69 246 (18.1)	1.25 (1.07-1.45)	1.31 (1.07-1.61)
Special health care needs												
Enrollment in care management services	62 750 (2.2)	0.90 (0.87-0.92)	2.23 (2.11-2.34)	44 590 (2.2)	0.80 (0.78-0.83)	2.08 (1.97-2.20)	11 643 (3.0)	0.80 (0.74-0.86)	1.73 (1.46-2.05)	6517 (1.7)	4.54 (3.69-5.59)	5.47 (4.19-7.14)

Abbreviations: NA, not applicable; OR, odds ratio; SDOH, social determinants of health.

^a^ For the race category, ORs were calculated between patients with minoritized race and patients with White race. This decision was made because the vast majority of patients were White, and the combined proportion of patients with minoritized race was less than 7%. The minoritized race category included American Indian or Alaska Native, Asian, Black or African American, Native Hawaiian/Pacific Islander, and other/unknown.

^b^ The other or unknown race category included those who selected “other” race or did not select a race.

^c^ Diabetes category included both uncomplicated and complicated diabetes.

^d^ Cardiovascular disease category included congestive heart failure, cardiac arrhythmias, and valvular disease.

^e^ Cancer category included lymphoma, metastatic cancer, and solid tumor without metastasis.

## Discussion

Our study found that patients screened in the ED were more likely to screen positive for SDOH needs, which is not surprising given utilization patterns. Patients with SDOH needs have limited health care access and are more likely to use the ED than primary care.^6^ Although primary care–based screening found lower SDOH needs relative to the ED, primary care may be better optimized to follow and ultimately address SDOH needs.^6^ As such and because overall percentages for screening were low, it may be best to screen for SDOH needs wherever feasible within a health system, thereby facilitating multiple points of entry for patients to access SDOH-related support. Surprisingly, encounters of patients with certain high-burden illnesses, such as cancer, were less likely to screen positive for SDOH needs, which may reflect heightened prior interaction with the study’s health system, as patients benefit from including system-specific presence of nurse navigators and cancer-specific care managers who can help address SDOH needs, leading to lower screening.

Limitations of this study include encounter-level rather than patient-level data. Implementation of the screening protocol in clinical practice varied in unobserved ways, especially during the COVID-19 pandemic, and may have contributed to selection bias. Although we included information about prior medical diagnoses, comprehensive past medical history and severity data were unavailable, limiting interpretation of findings related to clinical status. Additionally, less than 7% of patients encountered were not White, which limits study generalizability.

## Notes

Supplement 1. **eAppendix.** Screening Tools

Supplement 2.
Data Sharing Statement

## References

[1] Centers for Medicare & Medicaid Services, Department of Health and Human Services. Medicare program; hospital inpatient prospective payment systems for acute care hospitals and the long-term care hospital prospective payment system and policy changes and fiscal year 2023 rates; quality programs and Medicare promoting interoperability program requirements for eligible hospitals and critical access hospitals; costs incurred for qualified and non-qualified deferred compensation plans; and changes to hospital and critical access hospital conditions of participation; correction. Published online October 1, 2022. Accessed November 13, 2023. https://www.federalregister.gov/documents/2022/12/13/2022-26986/medicare-program-hospital-inpatient-prospective-payment-systems-for-acute-care-hospitals-and-the

[2] Horwitz LI, Chang C, Arcilla HN, Knickman JR. Quantifying health systems’ investment in social determinants of health, by sector, 2017-19. Health Aff (Millwood). 2020;39(2):192-198. doi:10.1377/hlthaff.2019.01246 32011928

[3] Andermann A. Screening for social determinants of health in clinical care: moving from the margins to the mainstream. Public Health Rev. 2018;39:19. doi:10.1186/s40985-018-0094-7 29977645 PMC6014006

[4] Intermountain Healthcare. Social determinants of health. 2020. Accessed May 30, 2023. https://intermountainhealthcare.org/ckr-ext/Dcmnt?ncid=529732182

[5] Knighton AJ, Savitz L, Belnap T, Stephenson B, VanDerslice J. Introduction of an area deprivation index measuring patient socioeconomic status in an integrated health system: implications for population health. EGEMS (Wash DC). 2016;4(3):1238. doi:10.13063/2327-9214.1238 27683670 PMC5019337

[6] Abir M, Hammond S, Iovan S, Lantz PM. Why more evidence is needed on the effectiveness of screening for social needs among high-use patients in acute care settings. Health Affairs Blog. Published online May 23, 2019. Accessed November 13, 2023. doi:10.1377/forefront.20190520.243444

