# Supplementary material for: Screening for Social Determinants of Health During Primary Care and Emergency Department Encounters
Source: JAMA Netw Open. 2023 Dec 19;6(12):e2348646. doi: 10.1001/jamanetworkopen.2023.48646 (PMC10731480; doi:10.1001/jamanetworkopen.2023.48646)
Supplement: Supplement 1. — eAppendix. Screening Tools [file jamanetwopen-e2348646-s001.pdf]

Supplemental Online Content

Vilendrer S, Thomas SC, Belnap T, et al. Screening for social determinants of health during primary care and emergency department encounters. *JAMA Netw Open*. 2023;6(12):e2348646. doi:10.1001/jamanetworkopen.2023.48646

eAppendix. Screening Tools

This supplemental material has been provided by the authors to give readers additional information about their work.

© 2023 Vilendrer S et al. *JAMA Network Open.*

Screening Tools

Between September 1, 2019, and December 1, 2020, Intermountain primary care clinics deployed SDOH screens in its adult and pediatric population, using PRAPARE Lite[1] for patients aged 6 and older and SEEK[2] for patients younger than 6.

The PRAPARE Lite assessment:

Life is not always easy. We want everyone to have a safe and healthy environment so we're asking everyone about challenges they face. If there's a problem, we may be able to help. Today or in the past year, have you or someone in your household had to go without any of the following when it was really needed?*

yes	no		yes	no	
☐	☐	Food	☐	☐	Medicine or prescriptions
☐	☐	Rent or mortgage payment	☐	☐	Medical services (such as a doctor or hospital)
☐	☐	Utilities (such as electricity, water, internet access, or phone)	☐	☐	Mental health services (such as treatment for anxiety or depression)
☐	☐	Feeling safe at home (such as safety from physical or emotional abuse, or theft)	☐	☐	Substance use disorder services (such as treatment for drug or alcohol misuse)
☐	☐	Transportation (such as a car or bus fare)			Other: ______________________
☐	☐	Education resources (school)			______________________
☐	☐	Dental care	☐	☐	I don't have a problem with these issues right now, or I choose not to answer this question

*This question comes from the national PRAPARE social determinants of health assessment protocol, developed and owned by the National Association of Community Health Centers (NACHC), in partnership with the Association of Asian Pacific Community Health Organization (AAPCHO), the Oregon Primary Care Association (OPCA), and the Institute for Alternative Futures (IAF). For more information, visit **www.nachc.org/prapare.**

[1] *Social Determinants of Health*. INTERMOUNTAIN HEALTHCARE; 2020. https://intermountainhealthcare.org/ckr-ext/Dcmnt?ncid=529732182

[2] Safe Environment for Every Kid Parent Questionnaire-R (SEEK PQ-R); 2017; https://ldh.la.gov/assets/oph/Center-PHCH/Center-PH/cshs/Developmental_Screening/SEEK.pdf

© 2023 Vilendrer S et al. *JAMA Network Open.*

Parent Questionnaire - R

Dear Parent or Caregiver: Being a parent is not always easy. We want to help families have a safe environment for kids. So, we're asking everyone these questions about problems that affect many families. If there's a problem, we'll try to help.
Please answer the questions about your child being seen today for a checkup. If there's more than one child, please answer "yes" if it applies to any one of them. This is voluntary. You don't have to answer any question you prefer not to. This information will be kept private, unless we're worried about your child's safety.

Today's Date: ___/___/____ Child's Name: ____________________________

Child's Date of Birth: ___/___/____ Relationship to Child: ____________________________

PLEASE CHECK

- ☐ Yes ☐ No Would you like us to give you the phone number for Poison Control?
- ☐ Yes ☐ No Do you need to get a smoke alarm for your home?
- ☐ Yes ☐ No Does anyone smoke at home?
- ☐ Yes ☐ No In the past 12 months, did you worry that your food would run out before you could buy more?
- ☐ Yes ☐ No In the past 12 months, did the food you bought just not last and you didn't have money to get more?
- ☐ Yes ☐ No Do you often feel your child is difficult to take care of?
- ☐ Yes ☐ No Do you sometimes find you need to slap or hit your child?
- ☐ Yes ☐ No Do you wish you had more help with your child?
- ☐ Yes ☐ No Do you often feel under extreme stress?
- ☐ Yes ☐ No Over the past 2 weeks, have you often felt down, depressed, or hopeless?
- ☐ Yes ☐ No Over the past 2 weeks, have you felt little interest or pleasure in doing things?

Thinking about the past 3 months

- ☐ Yes ☐ No Have you and a partner fought a lot?
- ☐ Yes ☐ No Has a partner threatened, shoved, hit or kicked you or hurt you physically in any way?
- ☐ Yes ☐ No Have you had 4 or more drinks in one day?
- ☐ Yes ☐ No Have you used an illegal drug or a prescription medication for nonmedical reasons?
- ☐ Yes ☐ No Other things you'd like help with today: ____________________________________

Please give this form to the doctor or nurse you're seeing today. We encourage you to discuss anything on this list with her or him. Thank you!

©2019, SEEK

© 2023 Vilendrer S et al. *JAMA Network Open.*
