# Supplementary material for: Screening for Social Determinants of Health During Primary Care and Emergency Department Encounters
Source: JAMA Netw Open. 2023 Dec 19;6(12):e2348646. doi: 10.1001/jamanetworkopen.2023.48646 (PMC10731480; doi:10.1001/jamanetworkopen.2023.48646)
Supplement: Supplement 2. — Data Sharing Statement [file jamanetwopen-e2348646-s002.pdf]

Data Sharing Statement

Vilendrer. Screening for Social Determinants of Health During Primary Care and Emergency Encounters in a Large Integrated Health System. *JAMA Netw Open*. Published December 19, 2023. doi:10.1001/jamanetworkopen.2023.48646

Data
Data available: No

Additional Information
Explanation for why data not available: Compilation of unique patient characteristics may make data potentially identifiable.
